# Effect of immunosuppressants on the parasite load developed in, and immune response to, visceral leishmaniasis: A comparative study in a mouse model

**DOI:** 10.1371/journal.pntd.0009126

**Published:** 2021-02-01

**Authors:** Lorena Bernardo, Jose Carlos Solana, Alba Romero-Kauss, Carmen Sánchez, Eugenia Carrillo, Javier Moreno

**Affiliations:** WHO Collaborating Centre for Leishmaniasis, National Center for Microbiology, Instituto de Salud Carlos III, Majadahonda (Madrid), Spain; Centro de Pesquisa Gonçalo Moniz-FIOCRUZ/BA, BRAZIL

## Abstract

The increasing use of immunosuppressants in areas where visceral leishmaniasis (VL) is endemic has increased the number of people susceptible to developing more severe forms of the disease. Few studies have examined the quality of the immune response in immunosuppressed patients or experimental animals with VL. The present work characterises the parasite load developed in, and immune response to, *Leishmania infantum-*induced VL in C57BL/6 mice that, prior to and during infection, received immunosuppressant treatment with methylprednisolone (MPDN), anti-tumour necrosis factor (anti-TNF) antibodies, or methotrexate (MTX). The latter two treatments induced a significant reduction in the number of CD4^+^ T lymphocytes over the infection period. The anti-TNF treatment was also associated with a higher parasite load in the liver and a lower parasite load in the spleen. This, plus a possibly treatment-induced reduction in the number of cytokine-producing Th1 cells in the spleen, indicates the development of more severe VL. Interestingly, the MPDN and (especially) MTX treatments provoked a greater presence of soluble *Leishmania* antigen-specific multi-cytokine-producing T cells in the spleen and a lower liver parasite load than in control animals. These results highlight the need to better understand how immunosuppressant treatments might influence the severity of VL in human patients.

## Author summary

Patients who are immunosuppressed are at greater risk of developing visceral leishmaniasis (VL) when infected with *Leishmania*. Prior infection with HIV has been traditionally associated with an increased risk of developing VL, but the use of immunosuppressants in the treatment of autoimmune disease has been linked to a higher incidence of VL in *Leishmania*-endemic areas. It is important to understand the influence these treatments have on *Leishmania* infection, paying special attention to how they affect the immune response mediated by IFN-γ-, TNF- and IL-2-producing T lymphocytes (such cells are necessary if an infection is to be resolved). Studies in this area require the use of murine models of VL. C57BL/6 mice infected with *Leishmania infantum* that received immunosuppressant treatment with methylprednisolone, anti-tumour necrosis factor antibodies, or methotrexate prior to and during infection, showed differences in their immune response, and in the parasite load developed in various organs. These results highlight the need to better understand how immunosuppressant treatments might influence the severity of VL in human patients.

## Introduction

Leishmaniasis is a neglected—though widely distributed—tropical disease caused by protozoan parasites of the genus *Leishmania* [1]. Visceral leishmaniasis (VL), the most severe form of the disease in which parasites invade different internal organs, is associated with high morbidity and mortality [2]. In the Mediterranean Basin, Central Asia and Brazil, *Leishmania infantum* is the species mainly responsible. Successful resolution of the infection requires the host to mount an adequate Th1-type immune response characterised by the production of IFN-γ, TNF and IL-2 by CD4^+^ and CD8^+^ T cells [3,4].

Being in an immunosuppressed state is major risk factor for developing severe VL. For example, co-infection with HIV increases by some 100–2320 fold the chances of developing active VL [5,6]. Indeed, HIV-associated immunosuppression has been identified as a major emerging challenge in the control of VL [5]. However, an increase in cases of VL has also been detected among recipients of solid organ transplants [7,8] and patients receiving immunosuppressants to treat autoimmune diseases such as rheumatoid arthritis, lupus erythematous and inflammatory bowel disease [9,10]. Ever more frequently used in these contexts, these drugs may increase the number of people susceptible to *Leishmania* infection as well as condition the severity of disease. For example, VL caused by both *L*. *infantum* and *L*. *donovani* has been reported in patients treated with methylprednisolone (MPDN) for rheumatoid arthritis, along with reactivated disease, likely due to the immunosuppression induced [11,12].

Tumour necrosis factor (TNF) is a key cytokine in the activation of macrophages infected by *Leishmania*, and it plays a central role in the development of a protective Th1 immune response [13]. Antagonists of TNF, such as anti-TNF antibodies, are also used in the treatment of autoimmune disease. After their bonding to the target molecule, the multiplication of amastigotes in infected macrophages is favoured, increasing the chances of developing clinical leishmaniasis—an outcome confirmed in animal models lacking the gene for TNF [14].

Methotrexate (MTX), an antagonist of folic acid, blocks the synthesis of purines and pyrimidines [15]. Commonly used to treat autoimmune diseases, its precise mechanism of action remains unknown, although it has been described to reduce the inflammation associated with these diseases [16]. It also reduces the multiplication of lymphocytes, and thus has a direct effect on immune system function [16]; it might, therefore, influence the development of an adequate immune response to *Leishmania* infection.

The effects of the above agents on the immune response to and severity of VL have never been examined comparatively in standard experimental models. However, the murine model of *L*. *infantum* infection allows the study of the immune response and of the mechanisms affecting protection against/susceptibility towards the development of VL [17]. This immune response is organ-specific; the infection is usually cleared from the liver in the first few weeks, but it becomes chronic in the spleen and bone marrow [18]. The present work characterises the parasite load developed in, and immune response to, *L*. *infantum-*induced VL in C57BL/6 mice that, prior to and during infection, received immunosuppressant treatment with MPDN, anti-TNF antibodies, or MTX.

## Methods

### Ethics statement

This work was approved by the Committee on Ethics and Animal Welfare of the *Instituto de Salud Carlos III* (CBA 04_2018, PROEX 072/18), and performed according to Spanish legislation for the protection of animals for experimentation and other scientific purposes (Royal Decree 53/2013, law 32/2007), which adheres to European Directive 86/609/EEC.

### Animals and parasites

The experimental animals were female C57BL/6 mice (Charles River, USA). The promastigotes used in experimental infections belonged to the JPC strain of *L*. *infantum* (MCAN/ES/98/LLM-724) and were obtained from the cold-stored *Leishmania* strain collection at the National Center for Microbiology (Majadahonda, Spain). Promastigotes were cultivated at 27°C in Novy-MacNeal-Nicolle (NNN) medium plus RPMI-1640 L-glutamine medium (Lonza, Switzerland) supplemented with 10% foetal bovine serum (Sigma, USA), penicillin (100 U/ml), and streptomycin (100 μg/ml) (Lonza, Switzerland).

### Immunosuppressants and infection

The mice were divided into four groups (N = 6 per group) and received either: 1) intraperitoneal (i.p.) PBS three times per week (control group); 2) subcutaneous (s.c.) MPDN 16 mg/kg (Sigma, USA) once per week [19]; 3) i.p. anti-TNF antibodies 20 mg/kg (Leinco Technologies, USA) twice per week [20], or 4) i.p. MTX 2.5 mg/kg (Sigma, USA) three times per week [21]. All treatments were administered in volumes of 0.1 ml. One week after beginning these treatments (end of Week 0), the mice were infected by injecting the tail vein with 1x10^7^ parasites in 100 μl PBS. Immunosuppressive treatment was maintained until the end of the experiment (end of Week 4).

### Sample processing

200 μl of blood were extracted from the submaxillary vein into a tube containing EDTA once per week to examine the immune response mounted to the infection. This blood was centrifuged at 500 *g* for 10 min to isolate the plasma. The remainder of the sample was used to analyse the populations of CD4^+^ and CD8^+^ T cells via flow cytometry (see below).

At the end of Week 4 the mice were euthanised and blood, liver, spleen and bone marrow (from the femur) samples collected.

The liver and spleen were homogenised on Falcon Cell Strainer nylon membranes (Thermo Fisher Scientific, USA) to obtain cell suspensions, which were then centrifuged at 800 *g* for 10 min in PBS at 4°C. The spleen cell fraction thus obtained was washed with RBC buffer (Invitrogen, USA) to eliminate any erythrocytes. The clean spleen cells were then re-centrifuged as above, and the pellet resuspended in 2 ml of RPMI. The liver cells obtained in the first centrifugation above were directly resuspended in 2 ml PBS. Bone marrow was obtained from the femur (cleared of muscle tissue) by flushing RPMI into the bone’s central cavity. The extracted cells were resuspended in 200 μL PBS. DNA was extracted from 200 μl suspensions of the above-prepared cells using phenol:chloroform:isoamyl alcohol (25:24:1) [22]. Extracted DNA was resuspended in 100 μl of distilled water, and quantified using the NanoDrop ND-1000 Kit (Thermo Fisher Scientific, USA).

### qPCR measurement of parasite load

Extracted *Leishmania* DNA was quantified using the LightCycler FastStart Kit (Roche Applied Science, Germany), using primers R223 and R334 for the *Leishmania* 18S ribosomal subunit [23]. A standard curve was then used to calculate the number of parasites from the DNA quantification results as previously described [24].

### Humoral response

Antibodies against parasite antigens in plasma were determined by ELISA. Nunc MaxiSorp plates (Thermo Fisher Scientific, USA) were carpeted with 2 μg/ml of soluble *L*. *infantum* antigen (SLA), obtained as previously described [25]. The plates were washed with 0.5% PBS-Tween20 and the wells blocked with a 3% BSA in 0.5% PBS-Tween20 blocking solution. Plasma samples were diluted 1/100 in blocking solution and the wells prepared to represent a 50% serial dilution series. After incubation (1 h at 37°C) the wells were washed in 0.5% PBS-Tween20 and incubated again for 30 min at 37°C with horse radish peroxidase-conjugated secondary antibodies, i.e., anti-IgG (conjugated high and low molecular weight chains), anti-IgG1, or anti-IgG2c (Nordic Mubio, Netherlands) diluted 1:2000 in blocking solution. Finally, the wells were washed again in 0.5% PBS-Tween20 and reactions revealed using the SIGMAFAST OPD Kit (Sigma, USA) according to the manufacturer’s instructions. The colorimetric reaction was stopped using 2N HCl. Absorbance was measured at 490 nm in a MultiskanFC device (Thermo Fisher Scientific, USA) and antibody titres determined as the inverse value of the last serial dilution in the above series in which the reactivity was greater than that of negative controls.

### Flow cytometry measurement of T lymphocyte populations

CD4^+^ and CD8^+^ T cells in blood were marked with APC CD8 (Clone 53–5.8) and PE CD4 (Clone GK1.5) anti-mouse antibodies (Biolegend, USA) for 30 min at 4°C in the dark. Erythrocytes were then eliminated using BD FACS Lysing Solution (BD Biosciences, USA). After centrifugation (800 *g* for 6 min), the cell pellet was washed and fixed in 2% formaldehyde. Analysis was then performed using a BD FACSCalibur flow cytometer (BD Biosciences, USA) and the results examined using FlowJo v7.6.5 software (FlowJo LLC, USA). Total CD4^+^and CD8^+^ T cells were obtained by multiplying the frequency value for each population by the number of leukocytes previously determined in the extracted blood using a Scill Vet ABC Plus automatic counter (Horiba Medical, Japan).

To determine T lymphocyte cytokine production, 5x10^6^ splenocytes/ml were stimulated for 6 h at 37°C with plate-bound anti-CD3 antibodies (10 μg/ml), soluble anti-CD28 antibodies (2 μg/ml) (eBioscience, USA), *L*. *infantum* SLA (25 μg/ml), or with culture medium alone (non-stimulated control). After 2 h of culturing, 10 μg/ml brefeldin A (Sigma, USA) were added to block the secretion of cytokines.

To determine their viability, the above splenocytes were then washed with PBS and incubated for 30 min with the reagent provided in the LIVE/DEAD Fixable Aqua Dead Cell Stain Kit (Thermo Fisher Scientific, USA). They were then washed in PBS + 1% FBS and incubated for 5 min with anti-mouse Fc Block (BD Biosciences, USA). Surface antigen detection was performed by adding PerCp-Cy5.5 anti-mouse CD3 (clone 145-2C11), BUV395 anti-mouse CD4 (clone GK1.5), FITC anti-mouse CD8 (clone 53–6.7), BV510 anti-mouse CD19 (clone 1D3) and BV510 anti-mouse CD14 (clone 123323) antibodies, incubating for 20 min. After washing off excess antibodies, the cells were fixed and permeabilized with BD Cytofix/Cytoperm (BD Biosciences, USA) and then washed with Perm/Wash buffer (BD Biosciences, USA). Intracellular cytokines were marked by incubating the cells for 30 min with PE-Cy7 anti-mouse IFN-γ (clone XMG1.2), AlexaFluor 647 anti-mouse TNF (clone MP6-XT22) and PE anti-mouse IL-2 (clone JES6-5H4) (BD, USA or BioLegend, USA) antibodies at 4°C in the dark.

The populations of different cytokine-producing T cells were determined using a BD LSRFortessa X-20 flow cytometer (BD Biosciences, USA) and the results processed using FlowJo v.7.6.5 software (FlowJo LLC, USA) (see S1 Fig). The results for each cytokine were represented as the difference between those of stimulated (anti-CD3/anti-CD28 or SLA) cells and non-stimulated (RPMI-treated) cells.

### Statistical analysis

All result distributions were subjected to Shapiro-Wilk testing of normality. Differences between groups were examined using the two-tailed Student t test (if distributions were normal) or Mann-Whitney U test (if they were not). Significance was set at p<0.05. All calculations were performed using GraphPad Prism v.7 software (GraphPad Software, USA). The results are representative of two independent experiments.

## Results

### Parasite loads in different organs

**Fig 1:**
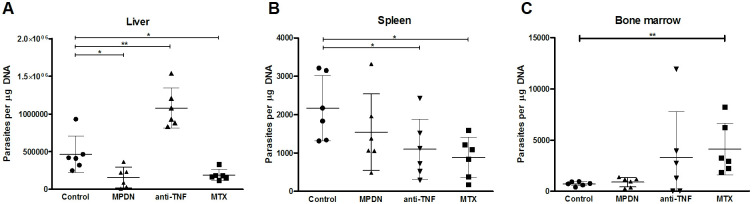
Parasite loads in the organs of *L*. *infantum*-infected mice treated with the different immunosuppressants. C57BL/6 mice (6 animals per group) were treated with PBS (control), MPDN (methylprednisolone), anti-tumour necrosis factor antibodies (anti-TNF) or MTX (methotrexate) one week before infection with 1x10^7^ *L*. *infantum* promastigotes. All animals were euthanized four weeks after infection and number of parasites per μg of DNA of liver (A), spleen (B) and bone marrow (C) were determined by qPCR. Results for each animal are shown, as well as the mean±standard deviation per group. *p<0.05, **p<0.01.

The anti-TNF-treated mice had a significantly higher liver parasite load than the control animals (p = 0.0018) (Fig 1A) but showed a significantly lower spleen parasite load than those same controls (Fig 1B).

The MTX-treated animals showed a lower parasite load than the controls in both the liver (p = 0.023) and spleen (p = 0.010), (Fig 1A and 1B), but a higher bone marrow parasite load (Fig 1C) (p = 0.0084).

As for the MTX-treated mice, the liver parasite load of the MPDN-treated animals was smaller than that seen in the control animals (p = 0.021), but no significant differences were seen between the spleen or bone marrow loads and those of the control animals.

### The number of T CD4^+^ cells circulating during infection is reduced by anti-TNF antibodies and methotrexate

**Fig 2:**
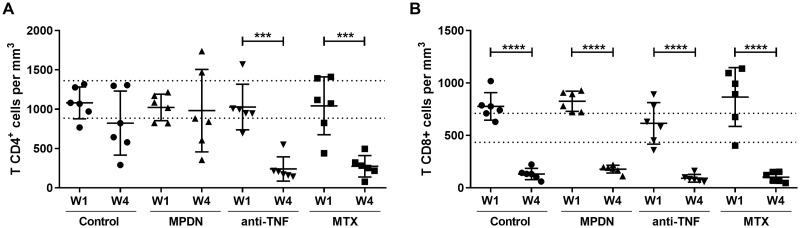
CD4^+^ and CD8^+^ T cell counts in immunosuppressant-treated mice infected with *L*. *infantum*. C57BL/6 mice (6 animals per group) were infected with 1x10^7^ *L*. *infantum* promastigotes one week after immunosuppressive treatment. Results show CD4^+^(A) and CD8^+^ (B) T cell counts in peripheral blood one (W1) and four (W4) weeks after infection for all animals in all treatment groups, plus the mean±standard deviation for each group. The normal count for CD4^+^ T cells is 1124±238 cells/mm^3^, and for CD8^+^ T cells 716±173 cells/mm^3^. ***p<0.001, ****p<0.0001. MPDN = methylprednisolone, anti-TNF = anti-tumour necrosis factor antibodies, MTX = methotrexate.

One week after infection (end of Week 1), the number of circulating CD4^+^ and CD8^+^ T cells remained unaffected by the immunosuppressant treatments (Fig 2); values for both fell within the range for naive mice of the same age (CD4^+^ T cells 1124±238 cells/mm^3^, CD8^+^ T 716±173 cells/mm^3^; determined in-house). However, at the end of the Week 4, the number of CD4^+^ T cells was significantly lower than at the end of Week 1, and indeed below normal values in both the anti-TNF- (76.6%; p = 0.0002) and MTX-treated (73.6%; p = 0.0007) mice (Fig 2A). This reduction coincided with an increase in the liver and bone marrow parasite loads (Fig 1A and 1C, respectively). However, all the treated animals, as well as the controls, showed a drastic reduction in the number of CD8^+^ T cells (p = 0.0001)—a result of the infection rather than any treatment (Fig 2B).

### Immunosuppressant treatment can influence the type and magnitude of the humoral immune response

**Fig 3:**
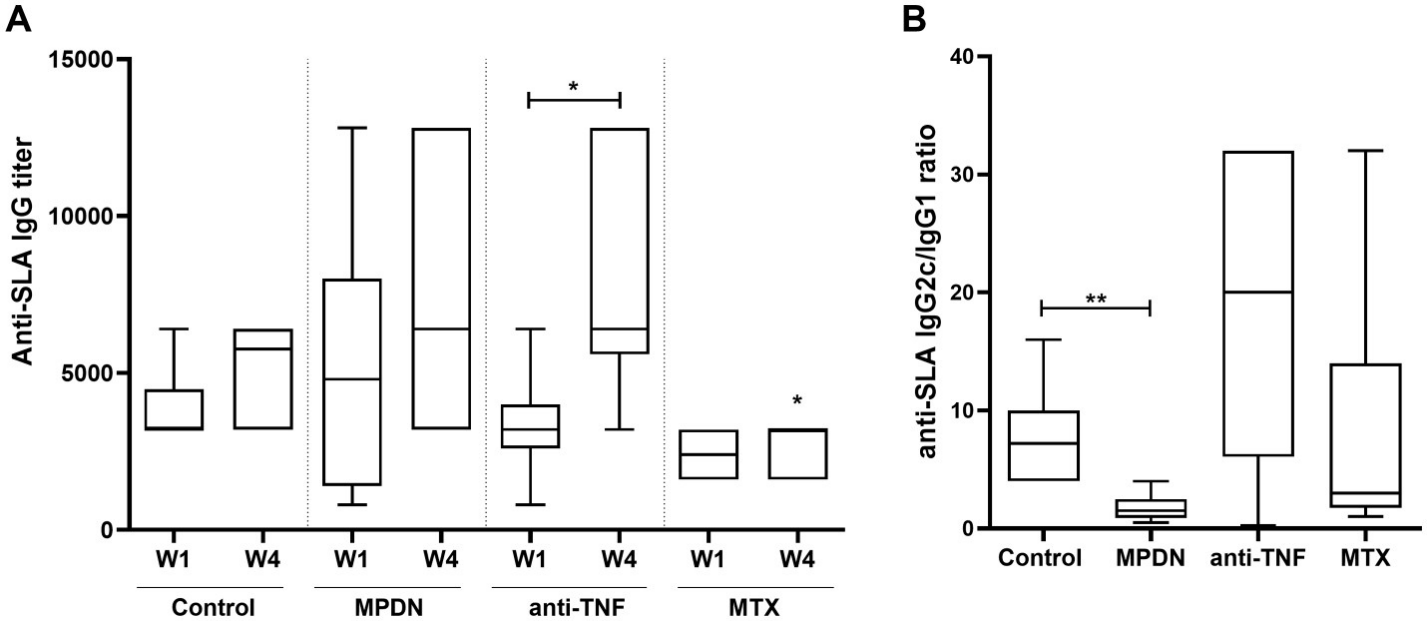
Humoral response of immunosuppressant-treated mice infected with *L*. *infantum* to soluble *Leishmania* antigen. C57BL/6 mice (6 animals per group) were treated with PBS (control), MPDN (methylprednisolone), anti-TNF antibodies or MTX (methotrexate) one week before infection with 1x10^7^ *L*. *infantum* promastigotes. Samples of sera of each animal were collected one (W1) and four (W4) weeks after infection to determine anti-SLA antibody titers by ELISA using limiting dilutions. The results are shown as median and whisker (min to max) plots for (A) anti-SLA total IgG antibodies one and four weeks after infection and (B) anti-SLA IgG2c/IgG1 subclasses ratio at W4. *p<0.05 ** p<0.001.

In response to *Leishmania* antigen, the *Leishmania*-specific total IgG titre increased from the end of Week 1 until the end of Week 4 (Fig 3A), but only significantly so in the anti-TNF-treated animals (p = 0.045). Interestingly, the MTX-treated animals returned an IgG titre significantly lower than that recorded for the control group by the end of Week 4 (p = 0.032).

No significant difference was seen between the IgG2c/IgG1 ratios of the anti-TNF-treated animals and the controls. The MPDN-treated mice showed a significantly less strong polarization towards the IgG2c type than did those of the control group (p = 0.0065) (Fig 3B). The latter result is not due to any smaller IgG2c titre in the MPDN-treated animals, but to a production of IgG1 antibodies far stronger than in any other group (S2 Fig).

### Immunosuppressant treatment can influence the cell-mediated immune response to *L*. *infantum*

In general, 10–20% of the splenocyte CD4^+^ and CD8^+^ T cells produced only TNF (S3 Fig) when challenged with α-CD3+α-CD28. In the control mice, only TNF-producing T cells were found, while the MPDN-, anti-TNF- and MTX-treated animals also showed CD4^+^ and CD8^+^ T cells that produced two (IFN-γ^+^TNF^+^ or IL-2^+^TNF^+^; up to 5% of T cells) or even three (IFN-γ^+^IL-2^+^TNF^+^; up to 4% of T cells in the MPDN-treated mice) of the examined cytokines (S3 Fig). The lymphocytes could thus be activated in all treatment groups, with TNF single-cytokine producers being the most common type generated.

**Fig 4:**
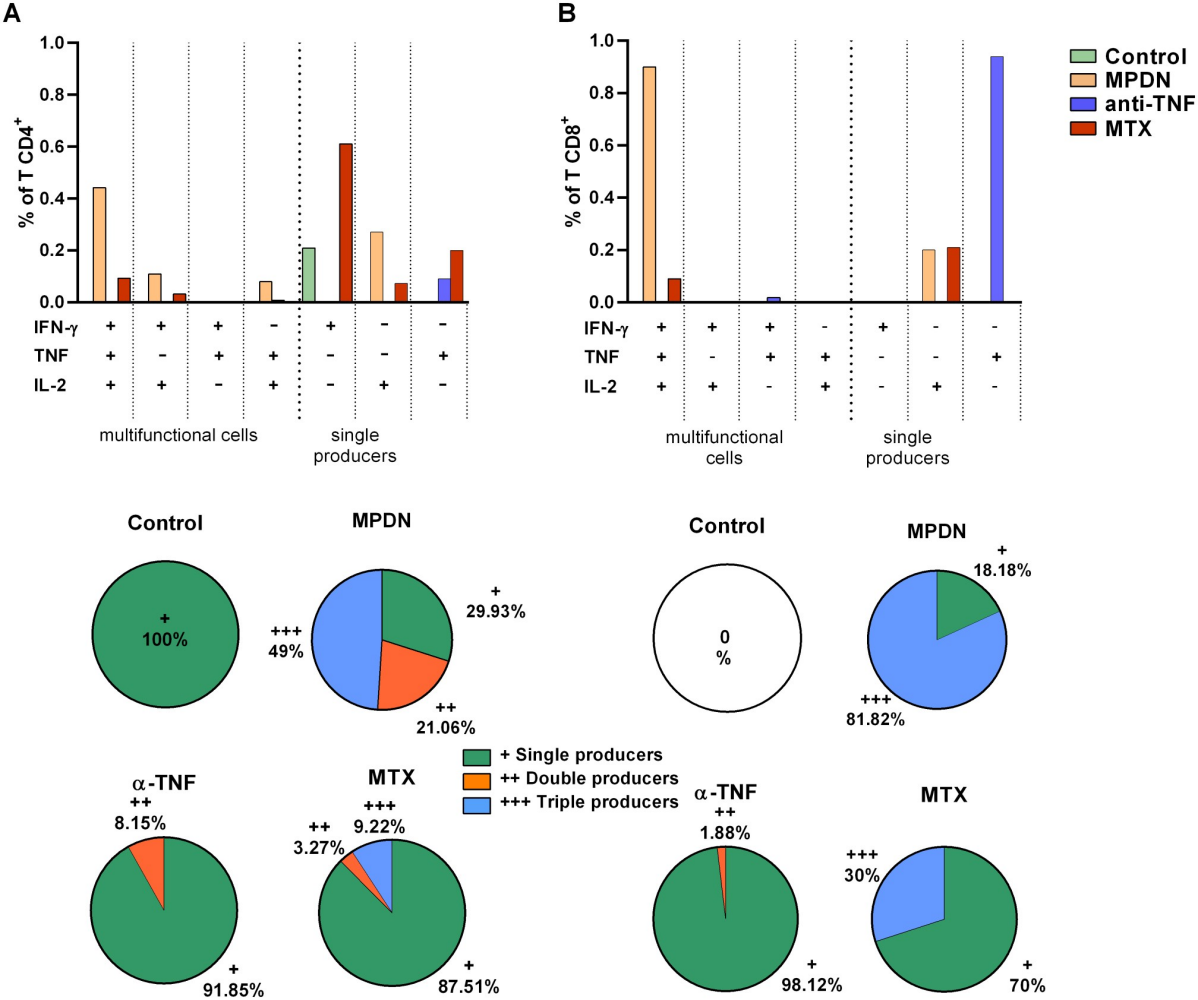
Cytokine-producing CD4^+^ and CD8^+^ T cells from the spleen of immunosuppressant-treated mice infected with *L*. *infantum*, following stimulation with soluble *Leishmania* antigen (SLA). C57BL/6 mice (n = 6 per group) were infected one week after starting drug treatment with 1x10^7^ *L*. *infantum* promastigotes and four weeks later the cytokine-producing capacity of T CD4^+^ (A) and T CD8^+^ (B) cells was determined by Boolean gating based on the expression of the cytokines IFN-γ, TNF and IL-2 (single-, double- and triple-cytokine producers). The upper graphs show the frequencies of CD4^+^ and CD8^+^ T cells producing one, two or three types of cytokine (IFN-γ^+^, TNF^+^ and IL-2^+^) in pooled splenocytes from each group of treated animals. The lower graphs show the percentage of total T cells that were IFN-γ^+^ or TNF^+^ or IL-2^+^single-cytokine producers (+), IFN-γ^+^TNF^+^, IFN-γ^+^IL-2^+^ or IL-2^+^TNF^+^ double-cytokine producers (++), and IFN-γ^+^TNF^+^IL-2^+^ triple-cytokine producers (+++) in each group of mice. MPDN = methylprednisolone, anti-TNF = anti-tumour necrosis factor antibodies, MTX = methotrexate.

The capacity of the CD4^+^ and CD8^+^ lymphocytes to respond to SLA differed between the treatment groups, and between them and the controls (Fig 4). The control animals generated only CD4^+^ IFN-γ^+^ producers (Fig 4A), while in the anti-TNF-treated mice this cytokine was produced only by a discrete population of CD8^+^ T cells (Fig 4B). In contrast, the MTX-treated animals generated mainly CD4^+^ IFN-γ^+^ producers, along with CD4^+^ TNF, CD4^+^ IL-2 and CD8^+^IL-2 single-cytokine producers, plus a discrete number of CD4^+^ and CD8^+^ IFN-γ^+^IL-2^+^TNF^+^ triple-cytokine producers. The MPDN-treated animals generated more CD4^+^ and CD8^+^ single-cytokine producers of IL-2, along with CD4^+^ IFN-γ^+^IL-2^+^ and TNF^+^IL-2^+^ double-cytokine producers. These animals also generated the greatest number of CD4^+^ and CD8^+^ IFN-γ^+^IL-2^+^TNF^+^ triple-cytokine producers (Fig 4A and 4B).

## Discussion

Anti-TNF antibodies, MPDN and MTX are among the immunosuppressants most often used to treat rheumatoid arthritis, lupus erythematous and inflammatory bowel disease [26]. Moreover, patients thus treated who live in *Leishmania*-endemic areas are at greater risk of infection by this parasite [27,28]. The present work compares the parasite load in, and immune response of, mice treated with these agents that were infected with *L*. *infantum*. The results show that these treatments influence the course of infection.

TNF plays a central role in the response to infection by *Leishmania*, promoting the activation of infected macrophages [29]. In the present model, the continued administration of anti-TNF antibodies led to an increased liver parasite load. The liver experiences the highest parasite loads of any organ in the first weeks of infection, but later these fall spontaneously [30,31]. The present results show that the administration of anti-TNF antibodies impedes this natural resolution. On the other hand, in this mouse model while granuloma formation and self-cure occurs in liver, the immune response against *Leishmania* begins to fail in the spleen and leads to uncontrolled parasite multiplication during the chronic phase of the infection [32]. Interestingly, although our analysis was performed before the chronic stage, we found in the spleen of control animals the highest parasite load. T cell exhaustion and tissue damage mediated by an excessive production of TNF (among other cytokines) lead to the remodelling of its architecture, an ineffective cellular response and compensatory mechanisms, such us IL-10 production, that promote parasite persistence [33]. Thus, anti-TNF treatment could reduce this host detrimental effect during the acute phase and delay the dysfunction of the spleen. Further experiments are necessary, including histological analysis and a continuous follow up of the disease progression to the chronic phase, in order to assess this question in mice models.

The anti-TNF animals also showed elevated titres of antibodies to *Leishmania*, a phenomenon that has been described as related to the presence of active VL in both patients and experimental animals [34,35]. In addition, the anti-TNF-treated mice showed a significant reduction in the number of circulating CD4^+^ T cells to below normal limits. As several authors have reported, leucopoenia regularly occurs with anti-TNF treatment [36,37], and in addition to that caused by VL could become a serious clinical problem. The anti-TNF treatment also influenced the functionality of the T cells in *L*. *infantum* infections; TNF antagonists are known to completely inhibit proinflammatory responses in patients [36,38]. Certainly, in TNF-knockout C57BL/6 mice infected with *Leishmania major*, the progression to VL is rapid, highlighting how important TNF is in the generation of cellular immunity against *Leishmania* [14,39]. Similarly, here we show for the first-time evidence in the liver of mice infected with viscerotropic species, which can be considered a site of acute self-resolving infection having similar features than asymptomatic individuals [40], of the risk of progressive visceral disease in otherwise asymptomatic infections. These findings are important since in recent years there has been an increase in the number of opportunistic *Leishmania* infections in patients treated with TNF antagonists, especially among those receiving monoclonal antibodies (Infliximab or Adalimumab) [41]. Clinicians should always weigh up the risk of the patient developing leishmaniasis before starting immunosuppressant treatment, but so far no guidelines are available to help in making such decisions.

Following infection, the MPDN-treated animals showed a reduction in the number of CD4^+^ T cells similar to that seen for the control group—but much less of a reduction than that seen in the other groups of treated mice. This can be related to the lower organ parasite loads in the MPDN and control animals (the resolution of *Leishmania* infections depends on a good CD4^+^ response [42]).

The humoral immune response to *Leishmania* is not protective; rather, it is a sign of advanced disease. Nevertheless, analysing the subtypes of IgG in experimental models is useful as a means of determining the kind of response underway [4,35]. In the present model, IgG2c antibodies were produced—a type related to the Th1 response. [43]. The MPDN-treated mice also produced IgG1 antibodies, which are associated with a Th2 response; this was not seen in any other group. However, the MPDN treatment also induced a clear Th1 response with T cells that produced different types of cytokine in the spleen. It has been amply described [4,42] that a specific Th1 response is necessary for resolving *Leishmania* infections; the cytokines produced, especially IFN-γ, mediate this protection [44]. The induction of this kind of response is essential if any immunotherapy or vaccine is to be successful [45]. Nevertheless, the immunobiology of VL is complex and resolution of the infection depends on an equilibrium between inflammatory and regulatory responses [42] and it seems that, despite a marked IgG1 antibody production, MPDN did not affect severely the anti-*Leishmania* response in the liver, spleen or bone marrow in a short-term infection.

The MTX-treated mice had a higher bone marrow parasite load than the mice of any other group. The bone marrow is sensitive to MTX, which suppress its function [46,47]. Interestingly, the spleen parasite load was lower in these mice than in either the controls or the other groups of treated animals. It has been reported that MTX has an antiparasitic effect against *Plasmodium vivax* and *Leishmania tropica* via its reduction of folate availability; by binding to parasite dihydrofolate reductase it reduces the availability of folate, which is essential to the survival of trypanosomatids [48,49]. However, it is important to note that *Leishmania* promastigotes has been described as having the potential to generate rapid resistance to methotrexate [50,51], which may trigger a worsening of the disease, especially in endemic areas of leishmaniasis. It is therefore necessary to monitor both aspects over time in order to better establish the relationship between *Leishmania* and the immunosuppressant MTX.

Patients treated with MTX appear to be at greater risk of VL than the immunocompetent population [52]. However, this treatment can be administered for over 60 months without greatly increasing this risk. In comparison, this risk becomes increased after just 17.5 months when TNF antagonists are administered [26,41].

In conclusion, the present results show that immunosuppressant agents can influence the immune response to *Leishmania* infection and the course of disease, even after a short immunosuppression period. The finding that treatment with anti-TNF antibodies can increase the liver parasite load is important given the many people treated with this agent in *Leishmania*-endemic areas [38,53]. Further analyses are necessary to evaluate if infection after long-term immunosuppression have even more harmful effects on the patient during parasite colonization and VL progression. Clinicians need to bear in mind the risk of leishmaniasis to patients thus treated, and how the dose employed might influence this. Likewise, the use immunological and molecular diagnostic tools to detect asymptomatic individuals and opportunistic infections in the follow up of immunosuppressed patients should be considered in endemic areas. Some parameters analysed in this work related with the T cell profile and the quality of the cellular response, in addition to those evaluated in future investigations, will help physicians to monitor these patients.

## Supporting information

S1 Fig Gating strategy used in the analysis of cell cultures. The first gate, FSC vs. SSC, was used to select the lymphocyte populations. Singlets were removed via the plot FSC-A vs. FSC-H. The CD3^+^ T cell population was selected by viability marking (LIVE/DEAD Fixable Aqua Dead Cell Stain Kit), and CD4^+^ and CD8^+^ populations then distinguished. IFN-γ, TNF and IL-2 production by each population was then determined using a Boolean gating.
(TIF)

S2 Fig Titre of IgG1 antibodies against soluble *Leishmania* antigen in immunosuppressed C57BL/6 mice infected with *L*. *infantum*. One week after starting immunosuppressive treatment mice (n = 6 per group) were infected with 1x10^7^ *L*. *infantum* promastigotes. Median and whisker (min to max) plots for IgG1 titre at the end of the first (W1) and fourth (W4) weeks after infection for animals in all treatment groups. *p<0.05 **p<0.001. MPDN = methylprednisolone, anti-TNF = anti-tumour necrosis factor antibodies, MTX = methotrexate.
(TIF)

S3 Fig Cytokine-producing CD4^+^ and CD8^+^ T cells from the spleen of immunosuppressant-treated mice infected with *L*. *infantum*, following stimulation with α-CD3/α-CD28 cocktail. C57BL/6 mice (n = 6 per group) were infected one week after starting drug treatment with 1x10^7^ *L*. *infantum* promastigotes and four weeks later the cytokine-producing capacity of T CD4^+^ (A) and T CD8^+^ (B) cells was determined by Boolean gating based on the expression of the cytokines IFN-γ, TNF-α and IL-2 (single-, double- and triple-cytokine producers). The upper graphs show the frequencies of CD4^+^ and CD8^+^ T cells producing one, two or three types of cytokine (IFN-γ^+^, TNF^+^ and IL-2^+^) in pooled splenocytes from each group of treated animals. The lower graphs show the percentage of total T cells that were IFN-γ^+^ or TNF^+^ or IL-2^+^single-cytokine producers (+), IFN-γ^+^TNF^+^, IFN-γ^+^IL-2^+^ or IL-2^+^TNF^+^ double-cytokine producers (++), and IFN-γ^+^TNF^+^IL-2^+^ triple-cytokine producers (+++) in each group of mice. MPDN = methylprednisolone, anti-TNF = anti-tumour necrosis factor antibodies, MTX = methotrexate.
(TIF)
