# Supplementary figures and images for: Effect of immunosuppressants on the parasite load developed in, and immune response to, visceral leishmaniasis: A comparative study in a mouse model
Source: PLoS Negl Trop Dis. 2021 Feb 1;15(2):e0009126. doi: 10.1371/journal.pntd.0009126 (PMC7877784; doi:10.1371/journal.pntd.0009126)

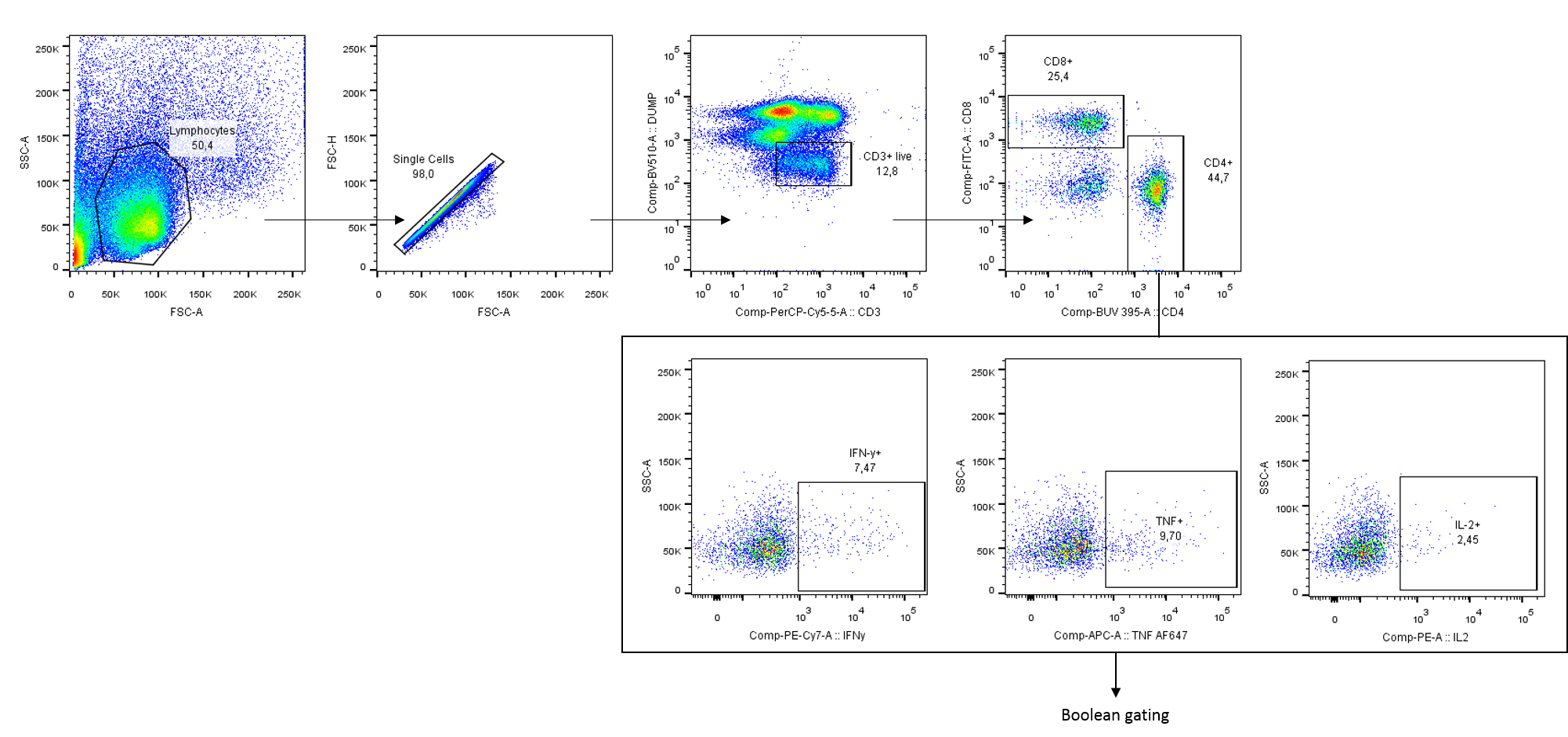

Supplement: S1 Fig — The first gate, FSC vs. SSC, was used to select the lymphocyte populations. Singlets were removed via the plot FSC-A vs. FSC-H. The CD3+ T cell population was selected by viability marking (LIVE/DEAD Fixable Aqua Dead Cell Stain Kit), and CD4+ and CD8+ populations then distinguished. IFN-γ, TNF and IL-2 production by each population was then determined using a Boolean gating. (TIF) [file pntd.0009126.s001.tif]

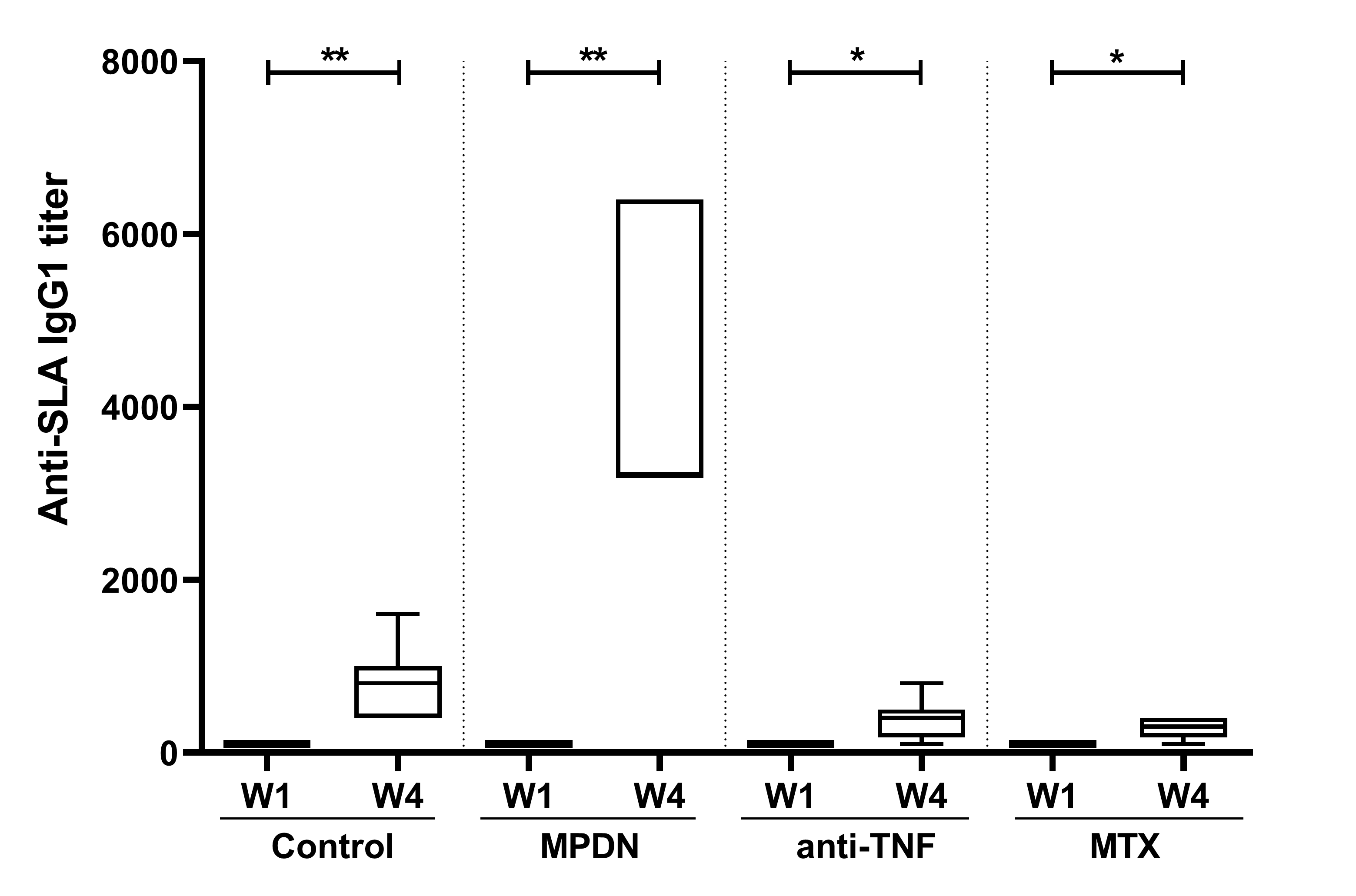

Supplement: S2 Fig — One week after starting immunosuppressive treatment mice (n = 6 per group) were infected with 1x10^7^ L. infantum promastigotes. Median and whisker (min to max) plots for IgG1 titre at the end of the first (W1) and fourth (W4) weeks after infection for animals in all treatment groups. *p<0.05 **p<0.001. MPDN = methylprednisolone, anti-TNF = anti-tumour necrosis factor antibodies, MTX = methotrexate. (TIF) [file pntd.0009126.s002.tif]

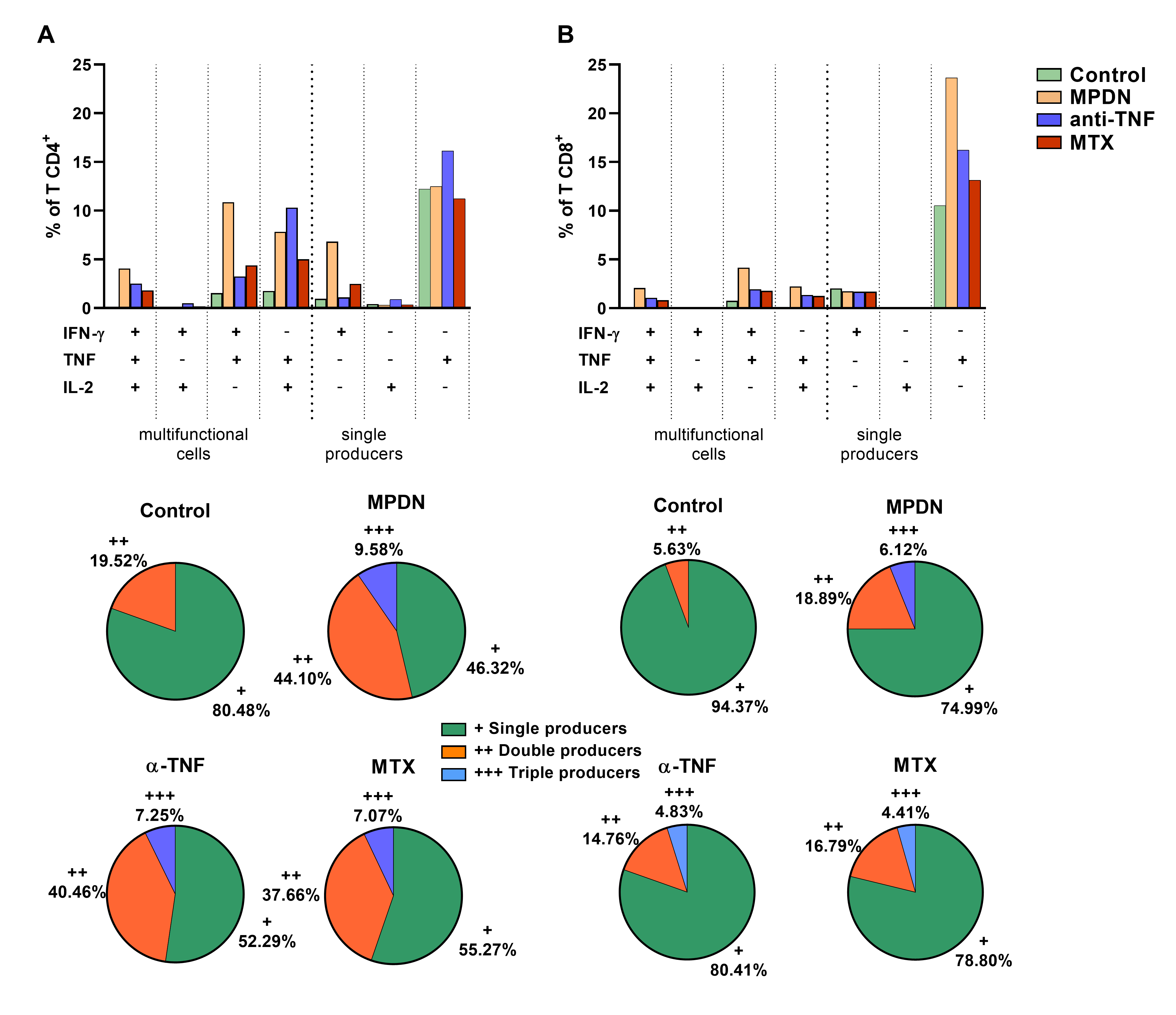

Supplement: S3 Fig — C57BL/6 mice (n = 6 per group) were infected one week after starting drug treatment with 1x10^7^ L. infantum promastigotes and four weeks later the cytokine-producing capacity of T CD4+ (A) and T CD8+ (B) cells was determined by Boolean gating based on the expression of the cytokines IFN-γ, TNF-α and IL-2 (single-, double- and triple-cytokine producers). The upper graphs show the frequencies of CD4+ and CD8+ T cells producing one, two or three types of cytokine (IFN-γ+, TNF+ and IL-2+) in pooled splenocytes from each group of treated animals. The lower graphs show the percentage of total T cells that were IFN-γ+ or TNF+ or IL-2+single-cytokine producers (+), IFN-γ+TNF+, IFN-γ+IL-2+ or IL-2+TNF+ double-cytokine producers (++), and IFN-γ+TNF+IL-2+ triple-cytokine producers (+++) in each group of mice. MPDN = methylprednisolone, anti-TNF = anti-tumour necrosis factor antibodies, MTX = methotrexate. (TIF) [file pntd.0009126.s003.tif]
